# Supplementary material for: Fast acting allosteric phosphofructokinase inhibitors block trypanosome glycolysis and cure acute African trypanosomiasis in mice
Source: Nat Commun. 2021 Feb 16;12:1052. doi: 10.1038/s41467-021-21273-6 (PMC7887271; doi:10.1038/s41467-021-21273-6)
Supplement: Supplementary file 1 — Supplementary Information [file 41467_2021_21273_MOESM1_ESM.docx]

**Supplementary Information**

**Fast acting allosteric phosphofructokinase inhibitors block trypanosome glycolysis and cure acute African trypanosomiasis in mice**

Iain W. McNae^#,1^, James Kinkead^#,1^, Divya Malik^#,1^, Li-Hsuan Yen^1^, Martin K. Walker^2^, Chris Swain^3^, Scott P. Webster^4^, Nick Gray^1^, Peter M. Fernandes^1^, Elmarie Myburgh^5^, Elizabeth A. Blackburn^1^, Ryan Ritchie^6^, Carol Austin^2^, Martin A. Wear^1^, Adrian J. Highton^2^, Andrew J. Keats^2^, Antonio Vong^2^, Jacqueline Dornan^1^, Jeremy C. Mottram^7^, Paul A.M. Michels^1^, Simon Pettit*^,2^, Malcolm D. Walkinshaw*^,1^

*corresponding authors: simon.pettit@selcia.com, [m.walkinshaw@ed.ac.uk](mailto:m.walkinshaw@ed.ac.uk)

^#^ These authors contributed equally to this work

^1^  Wellcome Centre for Cell Biology, School of Biological Sciences, University of Edinburgh, Michael Swann Building, Max Born Crescent, Edinburgh EH9 3BF, U.K.

^2^ Selcia Ltd., Fyfield Business and Research Park, Fyfield Road, Ongar, Essex CM5 0GS, U.K.

^3^ Cambridge MedChem Consulting, U.K.

^4^ Centre for Cardiovascular Science, College of Medicine and Veterinary Medicine, University of Edinburgh, U.K.

**^5^**York Biomedical Research Institute, Hull York Medical School, University of York, U.K.

^6^ Institute of Infection Immunity and Inflammation, College of Medical Veterinary Life-Sciences, University of Glasgow, U.K.

^7^ York Biomedical Research Institute, Department of Biology, University of York, U.K.

**Supplementary Methods**

**1. Protein Purification**

N-terminally His_6_-tagged T. brucei PFK (TbPFK) was expressed and purified as described previously (1). Briefly, a codon optimised TbPFK gene inserted in a pET28a vector was expressed in E. coli C41 cells. Protein was purified using a cobalt-charged HiTrap immobilised metal affinity chromatography (IMAC) column (GE Healthcare) at pH 8 followed by size exclusion using a Sephacryl™ S-200 16/60 column (GE Healthcare). The three human isoforms of L, M and P PFK were expressed and purified as described previously (1).

**2. Enzyme Assays**

**2.1 ADP-Glo™ assay for measuring PFK inhibition**

The ADP-Glo™ end-point assay kit manufactured by Promega (cat. No. V9102) was used to measure ADP produced in the phosphofructokinase reaction:

F6P + ATP ⮀ F16BP + ADP.

Following this kinase reaction, the addition of a reagent results in any unused background ATP being removed and the reaction is sequestered. A second reagent is then added to convert the remaining ADP to ATP which is coupled to the luciferase/luciferin reaction to produce light. ‘Ultrapure’ ATP and ADP from Promega were used to reduce background noise. All reagents additional to those provided in the Promega ADP-Glo™ kit were purchased from Sigma-Aldrich.

The assay buffer comprised 50 mM triethanolamine (TEA), 10 mM MgCl_2_, 5 mM KCl, 0.1% w/v bovine serum albumin (BSA), 0.005% Tween-20, 1% DMSO, pH 7.4. A 3-fold serial dilution of 250x inhibitor stocks was carried out in 100% DMSO. 2 µl of these dilutions was added to 198 µl assay buffer and mixed well using a multichannel pipette. A visual inspection of these dilutions was carried out to check for solubility at 1% DMSO. Concentration ranges of inhibitor compounds were 40 - 0.006 µM (final concentration, 1% DMSO). The PFK reaction in the presence of inhibitor was carried out using 10 µl of 1 µg/ml PFK (final concentration 0.4 µg/ml) which was added to 10 µl of the 1% DMSO inhibitor compound serial dilutions in a white, non-binding 96-well plate (Greiner BioOne 655904). CTCB-001 was used as a positive control at a final concentration of 5 µM. Assay buffer containing 1% DMSO was added instead of inhibitor compound for the negative control. The protein/inhibitor mixture was incubated for 10 minutes at room temperature (RT). The kinase reaction was started by adding a 5x ATP/F6P stock in assay buffer (final concentrations 0.5 mM F6P and 0.1 mM ATP). Plates were then sealed, centrifuged at 560 x g for 30 seconds and incubated at RT for 30 minutes. 25 µl of ‘ADP-Glo™ Reagent’ was then added to each well, mixed using a multichannel pipette and incubated at room temperature for 40 minutes. 50 µl of ‘Kinase Detection Reagent’ was added and incubated for 50 minutes at RT. The luminescence was recorded using a Spectramax© M5 Multi-mode Plate Reader (Molecular Devices) in single-point mode, with an integration time of 750 milliseconds. The luminescence values were converted to ADP concentration using a standard calibration curve for ADP. The resulting ADP concentrations were plotted as a function of inhibitor compound concentration using Kaleidagraph 4.0 software. The data were analysed using non-linear regression and a sigmoidal curve was fitted (Supplementary Figure 1) to determine the IC_50_. Kaleidagraph 4.0 uses the Levenberg-Marquardt algorithm in an iterative procedure to fit non-linear curves. The equation for the sigmoidal curve was fitted to the data using the positive control (100% inhibition of PFK – 5 µM CTCB-001) as the minimum constraint (m1) and the negative control (no inhibition of PFK - 1% DMSO) as the maximum constraint (m2) of the curve. The slope of the curve at the midpoint (m4) was set to 1.

$$ADP produced=m1+\frac{(m2-m1)}{(1+ \left( \frac{\left[ I \right]}{{IC}_{50}} \right)^{m4})}$$

Where, [I] = inhibitor concentration, m1 = minimum ADP produced, m2 = maximum ADP produced and m4 = slope of curve at midpoint (the Hill coefficient).

**2.2 Linked enzyme assay to determine inhibitor mode of action**

The mechanism of TbPFK inhibition was studied using a coupled enzyme assay in which production of ADP by TbPFK was coupled to the reaction of human M1 isoform pyruvate kinase (hPYK), which converts ADP and phosphoenolpyruvate (PEP) to ATP and pyruvate. The reaction is further coupled to lactate dehydrogenase (LDH), which converts pyruvate and NADH to lactate and NAD^+^, respectively. NADH conversion was monitored by measuring changes in NADH absorbance.

A 2.5x ‘assay mix’ was made containing 4.8 mM PEP, 1.2 mM NADH , 20 U/ml PYK, 33 U/ml LDH, 0 to 3mM ATP in assay buffer (10 mM MgCl_2_, 100 mM KCl, 50 mM TEA, 10% glycerol, 0.005% Tween-20, pH 7.4). For measuring activity 40 µl of assay mix was plated in a clear, non-binding 96 well plate (Greiner Bio-One cat. no. 655901) with 4-10 µl of the PFK sample (typically a 0.1 mg/ml stock) to be tested and 40 – 46 µl of assay buffer. The reaction was started upon addition of 10 µl of up to 6 mM F6P (0.6 mM final concentration). Absorbance at 340 nm was read at 20 second intervals for 10 minutes at 25^o^C in ‘kinetic mode’ on a Spectramax© M5 Multimode plate reader. The gradient of the linear section of raw plots of NADH absorbance over time were used to obtain initial velocities.

The reduction of V_max_ by CTCB-405 for both substrates indicates that the inhibitor is not competitive with either ATP or F6P which is consistent with the X-ray structures showing no steric overlap with the substrate binding sites and the CTCB inhibitor binding site. The increase in K_m_ for both ATP and F6P could be indicative of ‘mixed inhibition’ in which the inhibitor may bind to the enzyme whether or not the enzyme has already bound the substrate.

**2.3. ALDO/G3PDH/TIM linked enzyme assay for measuring PFK inhibition**

In the PFK/PYK/LDH assay, the linked PYK activity acts to convert the ADP produced by PFK back to ATP. As ATP is an inhibitor of human PFK (but not TbPFK) at concentrations higher than ~0.5 mM the regeneration of ATP is likely to affect the assay readout. An alternative linked assay was therefore developed using PFK/aldolase (ALDO)/glycerol-3-phosphate dehydrogenase (G3PD)/triosephosphate isomerase (TIM). In this assay F16BP produced by PFK is subsequently converted by aldolase into dihydroxyacetone phosphate (DHAP) and glyceraldehyde 3-phosphate (GA3P). TIM interconverts GA3P and DHAP, and DHAP is reduced to glycerol 3-phosphate by G3PD, alongside the oxidation of NADH to NAD^+^, which was measured by observing the change in UV absorbance at 340 nm for 10 minutes at 25° C using a Spectramax© M5 Multimode plate reader*.*

The assay buffer consisted of 50 mM TEA, 100 mM KCl, 10 mM MgCl_2_, 10% glycerol, 1 mM Tris(2-carboxyethyl)phosphine (TCEP), pH 7.4. Assay mix consisted of 5 U/ml G3PD (Sigma-Aldrich G6880), 2.5 U/ml ALDO (Sigma-Aldrich A2714), 25 U/ml TIM (Sigma-Aldrich T6258), and 1.25 mM NADH. ATP and F6P were obtained from Sigma-Aldrich (A2383 and F3627, respectively). All ligands and substrates (including ATP) were used at pH 7.4). 40 µl of assay mix, followed by 45 µl assay buffer, 4 µl of 0.1 mg/ml PFK and 1 µl of 100x serially diluted inhibitors were added to a non-binding, clear 96-well plate (Greiner Bio-One 655901) and incubated at RT for 10 minutes. The reaction was started by addition of 10 µl of 6 mM F6P (Supplementary Table 1).

**2.4 Selectivity of CTCB compounds against TbPFK over human PFK isoforms**

There are three isoforms of human PFK (1): PFK-M, PFK-L and PFK-P which have between 68% and 71% sequence identity. The smaller trypanosomatid PFKs (with monomer chain length of 487 amino acids) have approximately 20% sequence identity with the longer human isoforms (780 to 784 amino acids). Comparison of the X-ray structures of TbPFK (PDB 6QU4) with the X-ray structure of hPFK-P (PDB code 4XZ2) shows that the ATP and F6P substrate binding sites have some conserved features. A sequence alignment (Supplementary Figure 3) highlights those residues within 3.6 Å of ATP (red) and F6P (cyan). Of these 19 active site residues, 18 are absolutely conserved among the human PFKs and 12 are conserved with active site residues of TbPFK. In contrast, the allosteric pocket that binds the CTCB inhibitors comprises 12 residues within 3.6 Å from the bound ligand (coloured green in Supplementary Figure 3) and of these only two share identity with the hPFKs. This difference in sequence and structure between trypanosomatid and human PFKs is also clearly visualised in the Supplementary movies 1 and 2 which show the ligand binding pocket is not present in the X-ray structure of PFK-P (and given the almost completely conserved human isoform sequences at that region, the pocket will also be absent from PFK-M and PFK-L).

Inhibition of the CTCB lead compounds has also been experimentally tested against the three hPFK isoforms and in all cases no inhibition of the enzyme activity was observed up to 100 µM (the maximum concentration tested); see Supplementary Figure 4.

**3. Biophysical Binding Studies**

**3.1. Isothermal Titration Calorimetry (ITC)**

ITC results show that all of the CTCB compounds bind with an almost perfect 1:1 stoichiometry with TbPFK. The K_d_ values match well with the SPR results, shown here for CTCB-405 that has a K_d_ of 92 nM (ITC) compared with 82 nM (SPR).

ITC experiments were carried out in HBS buffer (10 mM HEPES, 150 mM NaCl, 0.005% surfactant p20, pH 7.4, 1% DMSO). Protein stocks were desalted (HiTrap 5 ml desalting column, GE Healthcare) with the above buffer to ensure minimal buffer mismatch between ligand and analyte. Compound titrations consisted of 16 injections with 180 second delay between injections and 750 rpm stirring at 25^o^C. All solutions were degassed prior to use. Binding stoichiometry is determined from the molar ratio of compound: protein at the equivalence point of the ITC curve (Supplementary Figure 5).

**3.2 Surface Plasmon Resonance (SPR)**

SPR studies use His_6_-TbPFK surfaces covalently attached to an NTA sensor chip. k_off_ values for CTCB compounds are in the range 0.5 s-1 and 3.5 s-1 (Supplementary Figure 6).

**3.3 Crystallisation and Structure Determination**

N-terminally His_6_-tagged T. brucei PFK (TbPFK) was expressed and purified as described previously (1). To obtain apo crystals, purified TbPFK in gel filtration buffer was concentrated to 6 mg/ml and crystallised via hanging drop at 290K. The well solution consisted of 9.5% PEG, 8000, 0.1 M sodium cacodylate pH 7.4. Crystals formed after 3 weeks. To obtain the complex with CTCB-12, apo-crystals were transferred to a 1µl drop containing well solution,1mM lCTCB-12 and 1% v/v DMSO for 10 minutes before being flash cooled directly in liquid nitrogen. Co-crystals of CTCB-360 were obtained from an initial screen using the Molecular Dimensions Morpheus crystallisation screen. The top hit contained 0.1 M carboxylic acids (sodium formate, ammonium acetate, sodium citrate tribasic dihydrate, sodium potassium tartrate tetrahydrate and sodium oxamate), buffer system 1 (0.1 M imidazole; MES monohydrate (acid)) and precipitant mix 3 (40% v/v glycerol; 20% w/v PEG 4000). Co-crystals were obtained by hanging drop at 290K with a protein concentration of 4.5 mg/ml. Protein was pre-mixed with CTCB-360 to a final concentration of 1 mM in 1% v/v DMSO. Crystals formed in 2 days Crystals of TbPFK with CTCB-405 were obtained by ligand exchange. Crystals grown in the presence of CTCB-360 were transferred to drops of well solution containing no ligands for 1 hour before being transferred to drops of well solution containing 1 mM ligand and 1% v/v DMSO for 10 minutes. For both CTCB-360 and CTCB-405 crystals were flash cooled directly in liquid nitrogen.

Data were collected on beamlines I03 (CTCB-12), I04 (CTCB-360) and I24 (CTCB-405) at the Diamond synchrotron radiation facility. Data were processed with XDS (3) and AIMLESS (4). Initial phases were obtained by molecular replacement using the program PHASER and the ATP containing structure 3F5M as the search model. Refinement was performed with the program REFMAC with manual adjustment using the program COOT, All programs used for structure determination and refinement are part of the CCP4 software suite (4). Data collection and refinement statistics are shown in Supplementary Table 2 and quality of electron density in Supplementary Figure 7.

**4. In vitro parasite killing assays**

**4.1. Trypanosome cultures**

The ‘in vitro’ parasite killing assay used the bloodstream-form of the non-human pathogenic subspecies T. b. brucei, strain Lister 427 (a gift from Professor Keith Matthews) which was cultured in HMI-9 medium containing 10% fetal bovine serum (FBS). A second cell line, T. b. brucei GVR35-Luc2 which constitutively expresses firefly luciferase (5) was grown in Modified HMI-9 plus 20% fetal calf serum (FCS) supplemented with 20% Serum Plus (Sigma) and 0.15 μg/ml puromycin as described previously (5). Both strains of trypanosomes were cultured in T-25 vented cap flasks at 37 °C and 5% CO_2_.

**4.2. T. b. brucei 427 growth inhibition assay**

Growth inhibition tests of bloodstream-form T. b. brucei Lister 427 were carried out using the LILiT (long incubation low inoculation test) method (6). The parasites were cultured in complete HMI-9 medium containing 10% FBS in 96-well plates (Greiner Inc.) with approximately 2500 trypanosomes per well. A 3-fold dilution series (in the range from 45 µM – 0.020 µM) of the inhibitor compounds were prepared in the plate; the maximal concentration of DMSO was 0.45%. After 72 hours of incubation at 37 °C and 5% CO_2_, 50 µl Alamar Blue (0.2% diluted with Dulbecco’s-phosphate buffered saline (D-PBS, Sigma) was added to each well and plates were incubated at 37 °C and 5% CO_2_ for 4 hours. Fluorescence was read on the BMG multiplate reader using an excitation wavelength of 530 nm and an emission wavelength of 590 nm.

**4.3. T. b. brucei Lister 427 time-to-kill assay**

The killing time of T. b. brucei Lister 427 was assessed using the CellTiter Glo 3D kit (Promega, Inc.) to measure trypanosome ATP levels as a real time indicator of viability. Compounds of interest were serially diluted from 16 µM to 0.5 µM in HMI-9 medium and added into a sterile white, flat bottom 96-well plate (Greiner Inc.). 2500 trypanosomes were added to each well. The plates were incubated at 37 °C and 5% CO_2_. At the end of each incubation period, CellTiter Glo 3D reagent was added to lyse the trypanosomes and the plates were incubated in the dark for 10 minutes at RT. Luminescence was measured at a wavelength of 580 nm using a BMG plate reader and percentage cell viability versus incubation time was determined.

**4.4. T. b. brucei GVR35-Luc2 time-to-kill assay**

The killing of T. b. brucei GVR35-Luc2 was assessed by adding D-luciferin to live cells resulting in the production of light in the presence of ATP and luciferase activity. Compounds of interest were serially diluted from 16 µM to 0.5 µM in Modified HMI-9 medium and added into sterile white, flat bottom 96-well plate (Greiner Inc.). 32,000 trypanosomes were added to each well. The plates were incubated at 37 °C and 5% CO_2_. At the end of each incubation, 200 µg/ml of D-luciferin was added to each well and incubated for 10 min. Luminescence was measured using a BMG plate reader and percentage cell viability versus incubation time was determined.

**4.5 The effect of glycerol concentration on the EC_50_ of CTCB compounds**

It has recently been shown that BSF T. brucei can also grow in culture without glucose but with glycerol (7) (8); see also Figure 1 of the main text of the paper. However, the concentrations of glycerol used in these studies were 5 mM and 10 mM, respectively. These concentrations are very high compared to the known free concentration of glycerol in blood for which values have been reported between approximately 50 and 200 µM - with values in adipose tissue where trypanosomes may also reside only slightly higher (see for example (9) (10)). The fact that the Km of glycerol kinase for glycerol is 0.44 mM (11), thus well above the blood levels, suggests that glycerol is unlikely to be a major energy and carbon source in situ. This notion is corroborated by our in vitro parasite killing experiments in the presence of increased amounts of glycerol in which we found minimal effects on EC_50_ values for the lead series (Supplementary Table 3).

**4.6 Wash-out Studies**

In vitro ‘wash-out’ studies were performed to determine the reversibility of the action of the CTCB compounds on trypanosomes, after a certain exposure time, and thus to assess whether the compounds have a trypanocidal or trypanostatic effect. These experiments were carried out with both the T. b. brucei Lister 427 and T. b. brucei GVR35-luc strains. Trypanosomes at a density of 500,000 cells/ml in culture medium were incubated with CTCB compounds at varying concentrations in 12-well plates in volumes of 1 ml for different periods of time at 37°C. Subsequently, the cultures were centrifuged for 5 min at 2600 x g, the cells washed by re-suspending the pellets in 1 ml medium without inhibitors, centrifuged again and re-suspended in 1 ml fresh medium. The cell suspensions were then incubated under regular culture conditions for 3 days and parasite growth monitored daily by light microscopy.

Data shown in Supplementary Figure 8 summarise the results of the wash-out experiments for CTCB-405 and CTCB-470 against the Lister 427 and GVR35 strains. The results for both strains are similar with no grow-back at concentrations of CTCB compounds greater than 2 to 4 µM with drug exposure longer than 4-6 hours.

**4.7 EC_50_ values against T. b. gambiense and T. b. rhodesiense**

CTCB-470 and CTCB-405 were tested at the Swiss Tropical Medicine Institute (STMI) against in vitro cultured T. b. gambiense and T. b. rhodesiense. Supplementary Table 4 shows a 2-fold improvement in killing for T. b. gambiense over T. b. brucei for both of the lead compounds that were tested.

**5. Pharmacokinetic Analyses**

**5.1 Cytotoxicity Testing**

To determine a cellular selectivity index, a HepG2 Cytotoxicity Assay was used. HepG2 cells (a human hepatocyte carcinoma cell line) were seeded into 96 well plates in EMEM media. Compounds were assessed for their effect upon cellular viability (6 point dose response curves). After 72h the numbers of viable cells were detected using Promega CellTitreGlo reagent to measure ATP using the luciferase/luciferin detection system. The selectivity index is calculated as the EC_50_ in the HepG2 assay divided by the EC_50_ determined in the T. b. brucei parasite killing assay.

**5.2. Inhibition of CYP450**

The compounds were investigated for their inhibitory effect on cytochrome P450 (CYP450) activity using five CYP450 isoforms (with fluorescent substrates in brackets): 3A4 (two substrates, BFC and DBF), 2D6 (AMMC), 2C9 (MFC), 2C19 (CEC) and 1A2 (CEC). Kits from BD Biosciences were used along with the method described by the manufacturer. In the first instance the compounds were tested at a single concentration (10 µM) to determine the % inhibition of CYP450 activity. Fluorescence was measured using a SpectraMax M5 plate reader. Less than 20% inhibition was found for all CYP isoforms except CYP2C19 which showed 45% and 64% inhibition by CTCB-470 and CTCB-405, respectively and CYP2D6 which showed 60% inhibition by CTCB-405. CTCB-508 did not inhibit any of the isozymes above 10%.

**5.3 Metabolic stability**

Compounds were tested for their metabolic stability using mouse and human liver microsomes (MLM and HLM). The compounds were tested at a single concentration of 5 µM. The samples were crashed with acetonitrile and put through protein precipitation plates. The eluent was analysed by LC/MS or LC/MS/MS.

**5.4 Plasma Protein Binding**

Compounds were tested for protein binding using mouse and human plasma at a single concentration of 10 µM and measured by equilibrium dialysis between plasma and buffer. The amount of parent was determined on both sides of the membrane after incubating for 4 hours at 37 °C. The samples were crashed with acetonitrile and put through protein precipitation plates. The eluent was analysed by LC/MS or LC/MS/MS. Output was percentage bound.

**6 Animal Studies**

**Ethical statement for animal models**

The experiments involving CD-1 mice were carried out in accordance with the United Kingdom Animals (Scientific Procedures) Act (1986) under Home Office regulations. Studies were performed in SPF facilities at the University of Edinburgh under licence PPL 70/8102 and at the University of Glasgow under PPL 60/4442. The experiments were approved by the local ethics committees of the respective universities.

**6.1. Pharmacokinetic studies in mice (outsourced to Pharmidex** <https://www.pharmidex.com/>)

CTCB-405 was administered to female CD-1 mice by IV bolus (1 mg/kg) by tail vein injection and oral dosing (5 mg/kg) at Pharmidex (UK). Blood samples were collected from terminal cardiac puncture at 0.08 (IV only), 0.25, 0.5, 1 (PO only), 2, 4, 8 and 24 hour time points. The whole blood was centrifuged to obtain plasma. Brains were also collected following cardiac perfusion from both sets of animals at 2 hours. Analysis of the biological samples was carried out by the company Selcia. Plasma proteins were precipitated by addition of PBS (1 volume) followed by 3 volumes of acetonitrile containing internal standard (verapamil). Following filtration through a protein precipitation plate the eluent was dried under nitrogen and re-suspended in mobile phase prior to analysis. Individual brains were homogenised in PBS and diluted to 10% w/v and divided into aliquots consisting of brain homogenate (1 volume, 0.3 ml) and 3 volumes (0.9 ml) of acetonitrile containing internal standard. Samples were centrifuged and supernatants removed and taken through a protein precipitation plate alongside an appropriate standard. Eluents were dried under nitrogen and re-suspended in mobile phase prior to analysis. Eluents were analysed by LC-MS/MS using a Phenomenex Gemini NX column (C18,30 x 2.1 mm, 3 micron). Analysis of the data was carried out using PK solutions software.

**6.2 Tolerance studies and in vivo pharmacokinetic studies**

Dosing regimens used in efficacy studies were first tested for tolerance in female CD-1 mice (University of Edinburgh; 6-8 weeks old). Animals were dosed orally by gavage with CTCB compounds following the dosing regimen for each individual experiment (n=5) as indicated. Compounds were administered in a mixture containing Cremophor EL: EtOH: D-PBS (30:10:60 v/v). Animals were monitored for up to 8 hours post-dosing. On the final day of dosing, 2 hours after the final dose, whole blood was harvested from three mice via cardiac puncture, under gaseous isoflurane anaesthesia, to obtain plasma for analyses. The remaining two mice were monitored for several days post dosing. To avoid coagulation, 100 μl of 2% sodium citrate was utilised in the syringe during the procedure and blood samples subsequently transferred to heparin coated microtubules. Blood samples were centrifuged at 800 x g for 10 minutes at room temperature. The supernatant containing the plasma was transferred to a fresh tube for determination of compound levels and further PK analysis.

**6.2.1 Predicted Drug Doses**

Modelling drug concentration in plasma based on the measured PK parameters (Supplementary Figure 10) suggests that multiple dosing would give sustainable plasma concentrations above the levels measured in wash-out experiments (Section 4.6) and consistent with killing of parasites in vivo. Experimentally measured drug plasma levels at 2 hours after dosing are over ~20% of the predicted concentrations (Supplementary Table 5).

**6.3 Efficacy Studies**

**6.3.1 Mouse model of Stage 1 HAT (Edinburgh)**

 T. b. brucei Lister 427 parasites (1 x 10^5^) were injected into a single donor female CD-1 mouse (University of Edinburgh; 6-8 weeks old) intraperitoneally in D-PBS containing 10 mM glucose. Parasites for subsequent Stage 1 HAT infection were obtained from whole blood via cardiac puncture, under gaseous isoflurane anaesthesia as described above.   Parasites (1 x 10^3^) harvested from the donor mouse were then injected into the peritoneum of female CD-1 mice (University of Edinburgh; 6-8 weeks old) in D-PBS containing 10mM glucose. One day after infection, animals were either left untreated (n=3) or dosed orally by gavage (n=5) with CTCB compound in Cremophor EL: EtOH : D-PBS (30:10:60 v/v). The veterinary trypanocidal drug pentamidine isethionate (2.5 mg/kg for 4 consecutive days after infection) was administered intraperitoneally as control (n=3) for parasite elimination. Mice were monitored daily for parasitaemia day 1 post-infection onwards. Trypanosomes were quantified in blood samples from the tail vein by microscopy as previously described (12). Animals were sacrificed on the day that parasites were first detected in blood samples. (Supplementary Figure 11)

**6.3.2 Mouse model of Stage 1 and Stage 2 HAT (Glasgow)**

Female CD-1 (Charles River UK; 6-8 weeks old) mice were infected with 3 × 10^4^ T. b. brucei GVR35-VSL2 bloodstream form parasites intraperitoneally. T. b. brucei GVR35-VSL2 expresses red-shifted luciferase and provides a better signal for imaging (13). Once infection was established, starting on day 7 for Stage 1 studies and day 21 for Stage 2 studies, mice were either left untreated (n=3) or dosed by oral gavage with CTCB compound in Cremophor EL: EtOH : D-PBS (30:10:60 v/v) (n = 6)**.**  Additional groups of mice (n=3) that received either a single intraperitoneal dose of diminazene aceturate (Sigma-Aldrich) at 40 mg/kg in sterile water or 0.1 ml melarsoprol gel (14) containing 3.6 mg melarsoprol, applied topically for 3 consecutive days, served as controls for Stage 1 and Stage 2 treatment studies, respectively.

Progression of T. b. brucei GVR35-VSL2 infection was monitored primarily by in vivo bioluminescence imaging of infected mice using an IVIS Spectrum (PerkinElmer) as described previously (5). For stage 1 studies, imaging of infected mice was performed before treatment on day 7 post-infection and in days following the treatment (days 8, 9 and 14). Imaging on groups of three mice was performed 12 minutes after intraperitoneal injection of 150 mg/kg D-luciferin (Promega) in PBS. For stage 2 studies, mice were imaged prior to treatment on day 21 and subsequently on days 22 and 23. On day 23, 24 hours after the final dose, mice were sacrificed by cervical dislocation and perfused with phosphate-buffered saline containing 15 g.L^−1^ glucose to allow ex vivo imaging of whole brains. To this end, 100 μl D-luciferin was administered onto the brain surface and imaging performed immediately thereafter.

Data obtained from bioluminescence imaging were analysed using Living Image Software (PerkinElmer). Consistent regions of interest (ROI) were used for whole-body images or head images to reflect bioluminescence in total flux (photons per second). For each ex vivo brain image, an oval shaped ROI was used to display the bioluminescence detected at the respective endpoints. Dependent on the stage of the experiment under consideration, parasites were also quantified in blood samples from the tail vein by microscopy either from day 7 or day 21 post-infection onwards. (See Supplementary Figure 12 and Supplementary Figure 13 which shows side head images).

**7 Chemical Synthesis**

**Preparation of CTCB-405** **1-[(3,4-Dichlorophenyl)methyl]-5-[2-(dimethylamino)ethyl] pyrrolo[3,2-c]pyridin-4-one**

**Preparation of 4-Chloro-1-[(3,4-dichlorophenyl)methyl]pyrrolo[3,2-c]pyridine**

A solution of 4-chloro-1H-pyrrolo[3,2-c]pyridine (9.85 g, 0.065 mmol.) in anhydrous N,N-dimethylformamide (250 ml) was cooled to 0°C, before adding sodium hydride (60% in oil, 2.85 g, 0.071 mmol.) portion-wise over 10 minutes. The reaction mixture was stirred at 0°C for 5 minutes, then at room temperature for 30 minutes. It was again cooled to 0°C before adding 3,4-dichlorobenzyl bromide (10.3 ml, 0.071 mmol.) in N,N-dimethylformamide (20 ml). The reaction mixture was warmed to RT and stirred for 2.5 hours.

The reaction mixture was treated with saturated aqueous ammonium chloride solution and then extracted with ethyl acetate (2x). The extract was washed with brine (3x) and then dried over magnesium sulphate, filtered and evaporated to give a colourless gum. The gum was purified on a silica column, eluting with 30% ethyl acetate, 70% iso-hexane and then dried under vacuum to give the title product as a white solid (16.9 g, 83%). ^1^H NMR (CDCl_3_  ppm) δ 8.07 (d, 1H, J = 5.7 Hz), 7.39 (d, 1H, J = 8.2 Hz), 7.19 (d, 1H, J = 2.1 Hz), 7.16 (d, 1H, J = 3.3 Hz), 7.05 (dd, 1H, J = 5.8, 0.8 Hz), 6.88 (dd, 1H, J = 8.3, 2.1 Hz), 6.72 (dd, 1H, J = 3.3, 0.8 Hz), 5.27 (s, 2H). LCMS (m/z) [M+H] 311, 313, 315.

**Preparation of 1-[(3,4-Dichlorophenyl)methyl]-4-methoxy-pyrrolo[3,2-c]pyridine**

A solution of sodium methoxide (33% in methanol, 170 ml) was added to 4-chloro-1-[(3,4-dichlorophenyl)methyl]pyrrolo[3,2-c]pyridine. The stirred mixture was heated at reflux for 5 hours and then cooled over an ice bath before adding saturated aqueous ammonium chloride solution. The mixture was extracted with dichloromethane (3x). The extract was filtered through a hydrophobic frit, evaporated and dried under vacuum to give the title product as a cream solid (16.6 g, 100%).^1^H NMR (CDCl_3_  ppm) δ 7.84 (d, 1H, J = 6.0 Hz), 7.36 (d, 1H, J = 8.3 Hz), 7.17 (d, 1H, J = 2.0 Hz), 6.99 (d, 1H, J = 3.1 Hz), 6.86 (dd, 1H, J = 8.26, 2.0 Hz), 6.79 (d, 1H, J = 6.0 Hz), 6.67 (d, 1H, J = 3.1 Hz), 5.23 (s, 2H), 4.09 (s, 3H). LCMS (m/z) [M+H] 307,309.

**Preparation of 1-[(3,4-dichlorophenyl)methyl]-5H-pyrrolo[3,2-c]pyridin-4-one**

A solution of 1-[(3,4-dichlorophenyl)methyl]-4-methoxy-pyrrolo[3,2-c]pyridine (16.6 g, 0.054 mol.) in acetonitrile (350ml) was prepared and iodo(trimethyl)silane (47.5 ml, 0.27 mmol.) was added. The stirred reaction mixture was heated at 40°C for 3 hours then cooled to RT. The solution was poured onto ice (ca. 1 litre) and was left to stand for one hour. The resultant precipitate was filtered off and then successively washed on the sinter with water, 10% aqueous sodium sulphite solution and diethyl ether (2x). The solid was dried in a vacuum desiccator to yield the title product as a cream solid (14.2g, 90%).^1^H NMR (DMSO d6 ppm) δ 11.3 (1H, s), 7.59 (d, 1H, J = 8.3 Hz), 7.50 (d, 1H, J = 2.0 Hz), 7.34 (d, 1H, J = 3.1Hz), 7.10-7.17 (m, 2H), 6.70 (d, 1H, J = 7.2 Hz), 6.64 (d, 1H, J = 3.1 Hz), 5.39 (s, 2H). LCMS (m/z) [M+H] 293, 295.

**Preparation of CTCB-405 1-[(3,4-Dichlorophenyl)methyl]-5-[2-(dimethylamino)ethyl] pyrrolo[3,2-c]pyridin-4-one**

A stirred mixture of 1-[(3,4-dichlorophenyl)methyl]-4-methoxy-pyrrolo[3,2-c]pyridine (1000mg, 3.4 mmol.) in dimethyl sulfoxide (17 ml) was prepared and powdered potassium hydroxide (763 mg, 13.6 mmol.) was added. The reaction mixture was stirred for 15 minutes before adding 2-chloro-N,N-dimethyl-ethanamine hydrochloride (735 mg, 5.1 mmol.). The reaction mixture was stirred at room temperature for 4.5 hours and then it was diluted with brine. The mixture was extracted with ethyl acetate (3x). The extract was washed with brine (3x) and then dried over sodium sulphate, filtered and evaporated. It was purified on a silica column using 2% methanol, 98% dichloromethane, then 3% methanol, 97% dichloromethane to give a white foam. The foam was triturated with a mixture of iso-pentane and diethyl ether to give a white solid. The solid was filtered off, washed with iso-pentane (2x) and then dried under vacuum to yield the title product as a white solid (784 mg, 63%).^1^H NMR (CDCl_3_  ppm) δ 7.41 (d, 1H, J = 8.3 Hz), 7.19 (d, 1H, J = 2.0 Hz), 7.07 (d, 1H, J = 7.4 Hz), 6.86-6.92 (m, 3H), 6.20 (d, 1H, J = 7.4 Hz), 5.16 (s, 2H), 4.12 (t, 2H, J = 6.7 Hz), 2.66 (t, 2H, J = 6.7 Hz), 2.32 (s, 6H). LCMS (m/z) [M+H] 364, 366.

**Preparation of CTCB-470** **1-[(3-Bromo-4-chlorophenyl)methyl]-5-[2-(dimethylamino)ethyl] pyrrolo[3,2-c]pyridin-4-one**

CTCB-470 was prepared in a similar manner by using 3-bromo-4-chlorobenzyl bromide instead of 3,4-dichlorobenzyl bromide. ^1^H NMR (CDCl_3_  ppm) δ 7.4 (d, 1H, J = 8 Hz), 7.35 (m, 1H), 7.05 (d, 1H, J = 8 Hz), 6.9 (d of d, 1H, J = 2, 8 Hz), 6.85 (m, 2H), 6.175 (d, 1H, J = 8 Hz), 5.15 (s, 2H), 4.1 (t, 2H, J = 7 Hz), 2.65 (t, 2H, J = 7 Hz), 2.3 (s, 6H)

**Preparation of CTCB-508** **1-[(3-Bromo-4-fluorophenyl)methyl]-5-[2-(dimethylamino)ethyl] pyrrolo[3,2-c]pyridin-4-one**

CTCB-508 was prepared in a similar manner by using 3-bromo-4-fluorobenzyl bromide instead of 3,4-dichlorobenzyl bromide. ^1^H NMR (CDCl_3_  ppm) δ 7.3 (m, 1H), 7.05 (m, 2H), 6.9 (m, 2H), 6.2 (d, 1H, J = 7 Hz), 5.15 (s, 2H), 4.1 (t, 2H, J = 7 Hz), 2.675 (t, 2H, J = 7 Hz), 2.3 (s, 6H)

Details of the synthesis of additional active compounds with the same mode of action are available in a published patent (15)

**8 Determination of pKa values**

pKa and AlogP values for selected CTCB compounds were determined experimentally by the company Pharmorphix ([www.matthey.com](http://www.matthey.com/)) and calculated pKas were determined using ChemAxon.software (https://chemaxon.com).

A comparison of the calculated values for CTCB-405 and CTCB-531 is given in Supplementary Table 6 and calculated pKa values for other selected CTCB compounds have been added to Figure 2 of the main paper.

**Supplementary Figures**


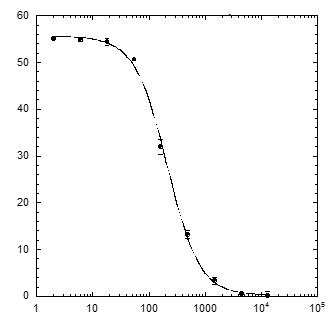


m2

ADP produced

Slope = m4

IC_50_

m1

Log [CTCB-405]

**Supplementary Figure 1.** **Typical inhibition curve for the CTCB series of inhibitors.**

Curves were fitted to the 4-parameter logistic equation where, [I] = inhibitor concentration, m1 = Minimum ADP produced, m2 = maximum ADP produced and m4 = slope of curve at midpoint (the Hill coefficient). For each data point n=2 independent experiments were carried out on the same plate in 2 separate columns. Data are presented as mean values +/- standard deviation**.** Source data are provided as a Source Data file.


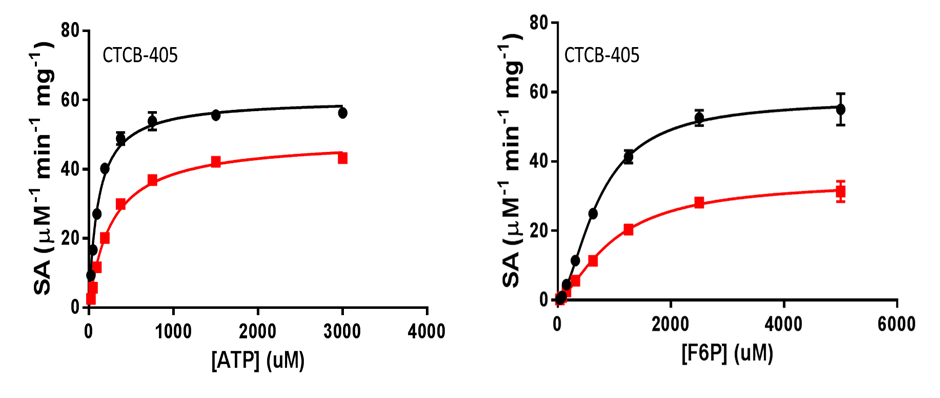


**Supplementary Figure 2. Michaelis-Menten curves showing non-competitive inhibition by CTCB-405 with respect to both ATP (left) and F6P (right).** Specific activity of TbPFK (µM/min/mg) is shown on the ordinate axis. For each data point n=2 independent experiments were carried out on the same plate in 2 separate columns. Data are presented as mean values +/- standard deviation**.**Source data are provided as a Source Data file.

Black curves: activity of TbPFK in the absence of inhibitor, red curves: in the presence of 200 nM CTCB-405.The titration of ATP (left) from 0 to 3 mM was in the presence of 10 mM F6P and the titration of F6P from 0 to 6 mM F6P (right) was in the presence of 5 mM ATP. The sustained suppression of V_max_ even at very high substrate concentrations shows CTCB-405 is not competitive with either substrate.


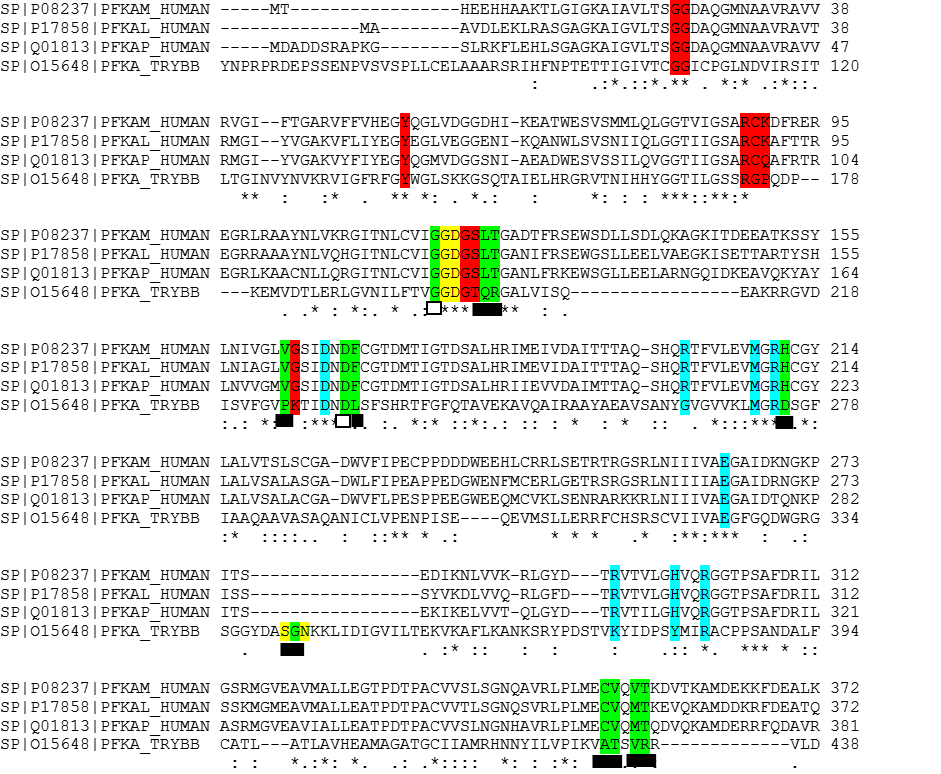


**Supplementary Figure 3. Sequence alignment of the N-terminal portion of human PFK-L, PFK-M, PFK-P and TbPFK.**

Residues within 3.6Å of ATP (taken from TbPFK structure 3F5M) are highlighted red,

residues within 3.6Å of F6P (taken from human structure hPFK-P 4XZ2) are highlighted cyan,

residues within 3.6Å of either CTCB-360 or CTCB-405 are highlighted green.

Residues highlighted yellow are involved in both ATP and CTCB-360/405 binding.

Black bars highlight the lack of identity between residues making up the allosteric pocket.


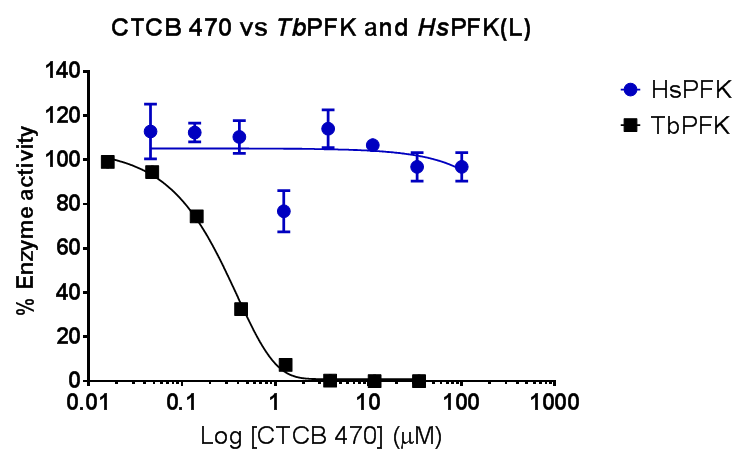


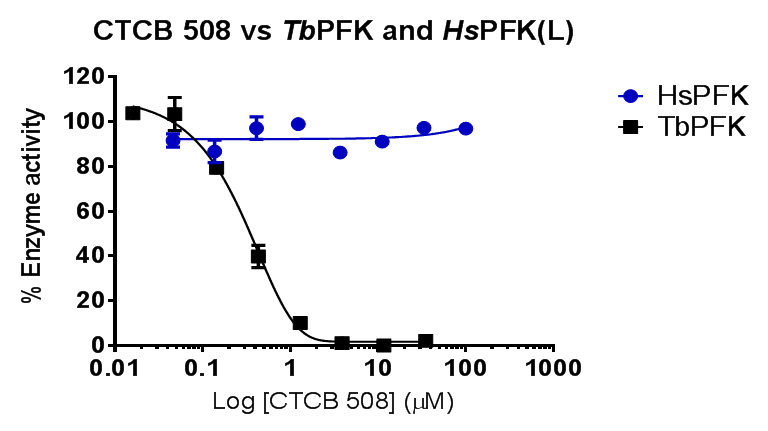


**Supplementary Figure 4. Titrations of CTCB-508, and CTCB-470 against hPFK-L and TbPFK using the aldolase/TIM linked enzyme assay as described in Section 2.3.**  For each data point n= 2 independent experiments were carried out on the same plate in 2 separate columns. Data are presented as mean values +/- standard deviation. Source data are provided as a Source Data file.


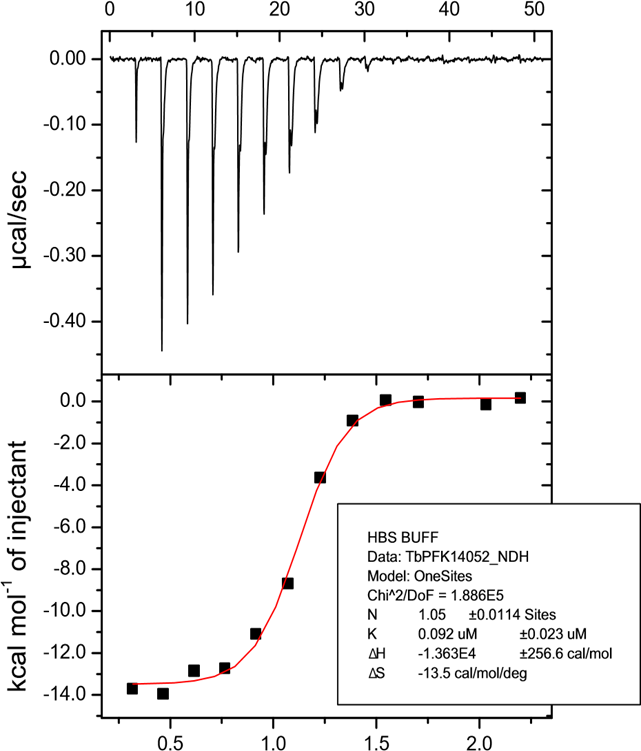


**Supplementary Figure 5. CTCB-405 binds TbPFK in a 1:1 stoichiometry as measured by ITC.**

ITC trace of titration of 150 µM CTCB-405 into 12.8 µM TbPFK at 25 °C. Top panel shows raw ITC trace for each injection, starting from highest to lowest injections of compound (left to right). Bottom panel shows integrated data of each injection (Kcal/mol) (Y-axis) against the molar ratio (X axis) with non-linear regression (one-site model) fitted. Source data are provided as a Source Data file.

**Supplementary Figure 6. SPR sensorgram for CTCB-405 titration against TbPFK.**

Active N-terminally His_6_-tagged TbPFK surfaces were generated using a His-tag capture and coupling method described previously (2). The surface density on the SPR chip = 1500 RU. A 1:1 binding model has been fitted to each sensorgram trace (black). All measurements were at 25^o^C. The sensorgram shows fast on rates and slow off rates, with the response reaching equilibrium at the higher concentrations. CTCB-405 completely dissociates back to baseline. Source data are provided as a Source Data file.

**
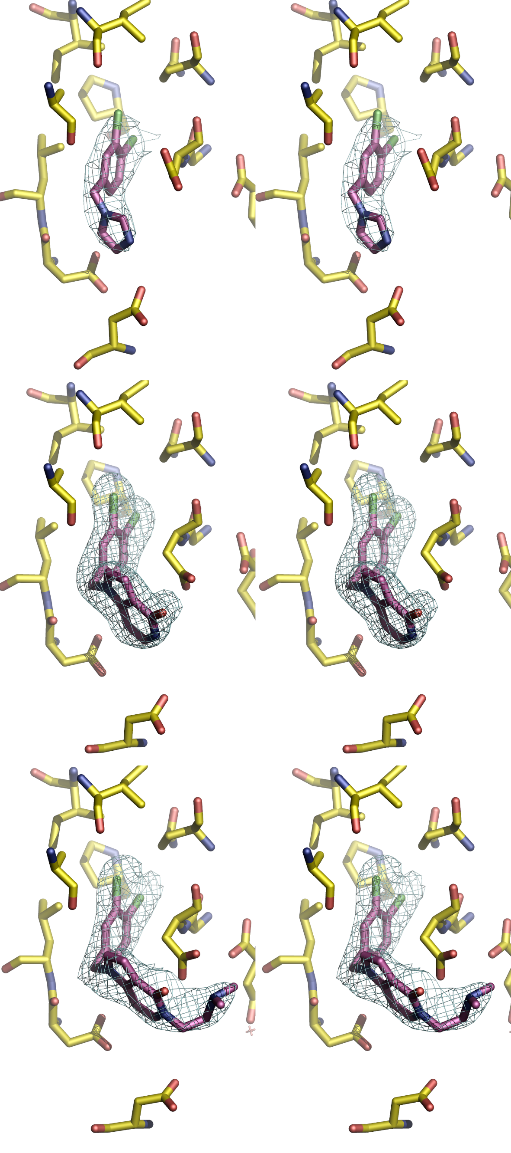
**

**Supplementary** **Figure 7.** Walleye stereo drawing of the 2|Fo|-|Fc| electron density contoured at 1 sigma round the ligands CTCB-12 (PDB 6QU5, top), CTCB-360 (PDB 6QU3, middle) and CTCB-405 (PDB 6QU4, bottom).

**Supplementary Figure 8.** **Wash out studies showing the results of treating Tbb Lister427 and TbbGVR35 with CTCB-405 and CTCB-470.** The red squares indicate that no parasites were observed after three days in drug free medium.

**
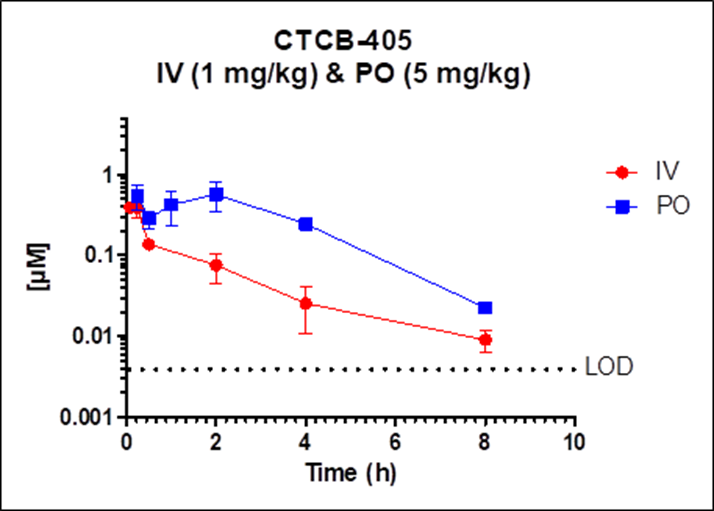
**

**Supplementary Figure 9. Concentration- time profile of CTCB-405 in plasma following administration of an IV bolus of 1 mg/kg or an oral dose of 5 mg/kg in mice.**

Data are mean +/- SD, n=3 at each time point. LOD is limit of detection. Source data are provided as a Source Data file.


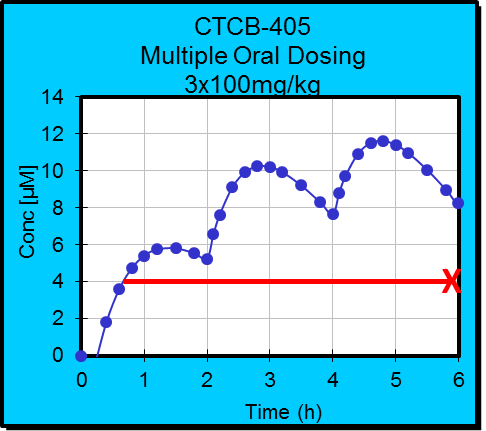


**Supplementary Figure 10.** **Predicted plasma concentrations for CTCB-405 for a multiple dosing regimen** (PK solutions 2.0, Noncompartmental PK data analysis, Summit Research Services, PK and Metabolism Software).


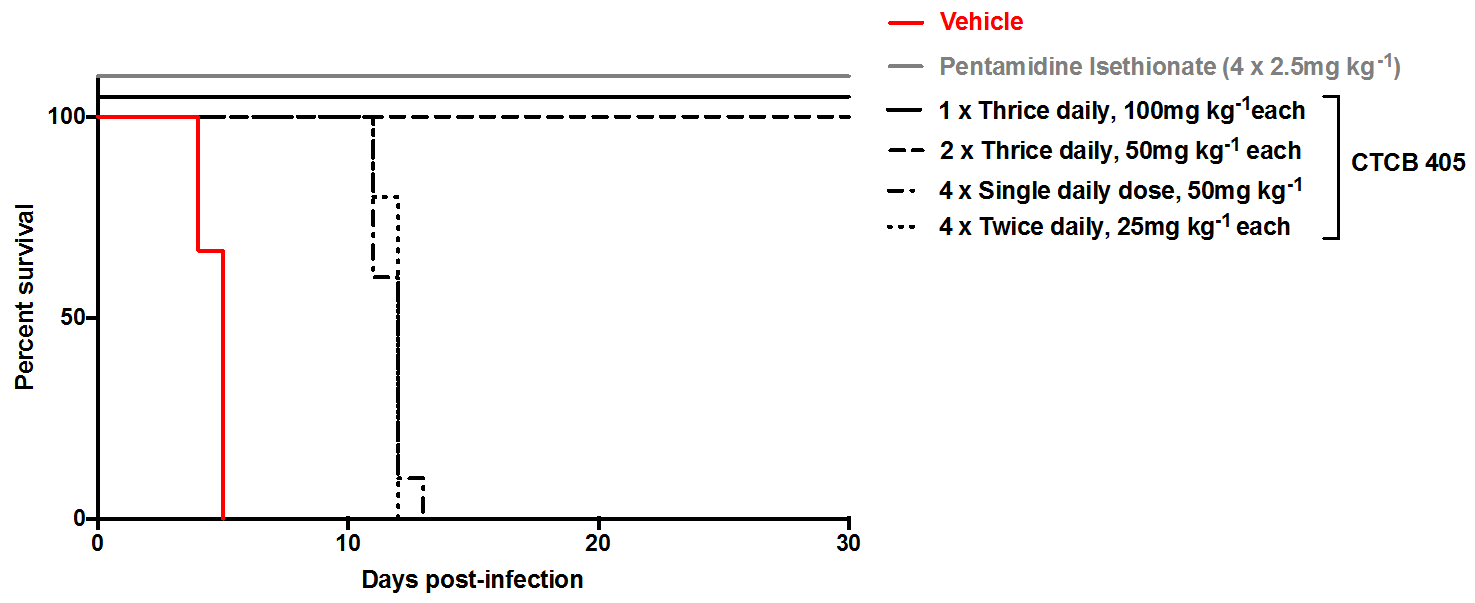


**Supplementary Figure 11.** **Dose response and parasite clearance following treatment with CTCB-405.**

Kaplan-Meier survival curves demonstrating progression of CTCB-405 oral dosing regimens in T. b. brucei Lister 427 infected mice. Single doses of 50 mg/kg (patterned, black; n=5) over four consecutive days delay parasitaemia up to 12 days post infection. Split dosing involving two daily doses at 25 mg/kg (dotted, black; n=5) delivered 4 hours apart also prolongs survival but does not improve efficacy. Administration of three 50 mg/kg doses 2 hours apart over 2 days (dashed, black; n=5) results in parasite clearance. Cure can also be achieved following one day dosing; parasites are undetectable in mice treated with three doses of 100 mg/kg 2 hours apart (solid, black; n=5), even after 30 days post-infection (regarded as cure for Stage 1 models). Control mice were administered either vehicle only (solid, red; n=3) or four single daily doses of 2.5 mg/kg pentamidine isethionate intraperitoneally (solid, grey; n=3). Mice were culled upon detection of first visible parasites in blood smears.

**Supplementary Figure 12. CTCB compounds cross the blood brain barrier and result in a reduction in parasitaemia in a pleiomorphic model of HAT infection** Mice infected with T. b. brucei GVR35 were treated topically with 3 doses of 3.6 mg melarsoprol over 3 days (n=3) or orally with 6 doses of CTCB compounds (CTCB-470 or CTCB-508) at 100 mg.kg^-1^ over 2 days (n=6). (Melarsoprol is a toxic anti-stage 2 HAT drug used as a positive control). Whole body bioluminescence (total flux in photons per second, above image) and blood parasitaemia (in parasites.ml^-1^, below image) for 3 representative mice from each group is shown in Figure 7 of the main text of the paper. a) At day 23 mice shown in Figure 7 of the paper were perfused with saline; brains were removed, soaked in luciferin and imaged to detect bioluminescent parasites. Images of brains are shown in a high sensitivity scale. b) Bioluminescence of brains imaged ex vivo on day 23 (shown in a and Figure 7c of the paper). (Supplementary Figure 13 shows side head images). Source data are provided as a Source Data file.


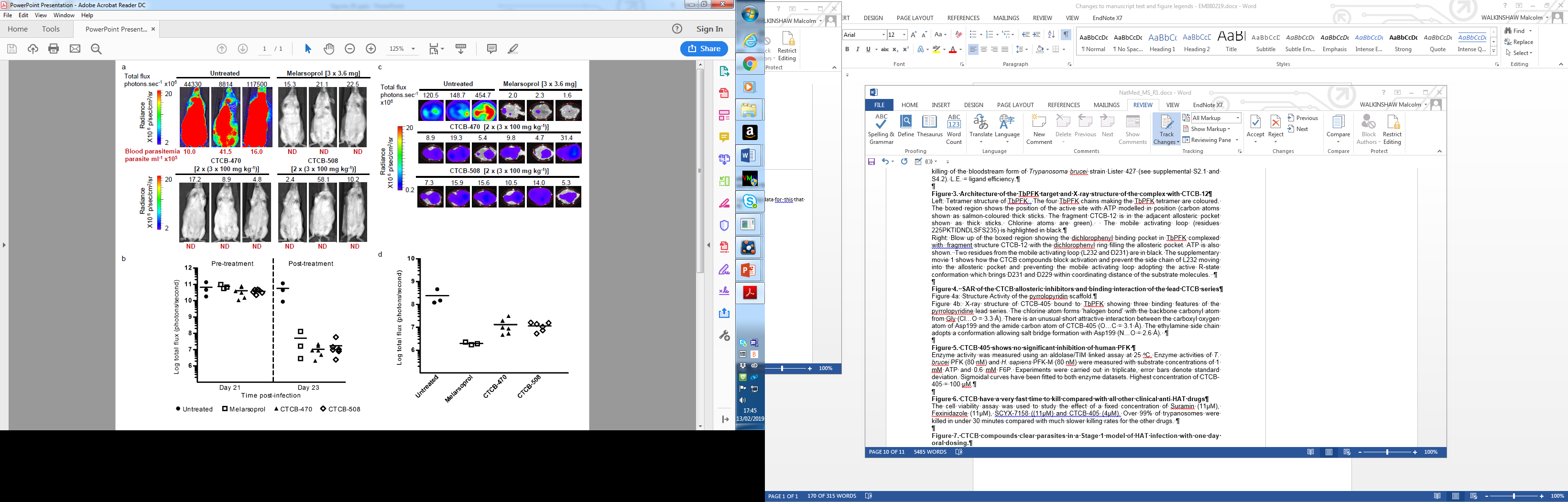

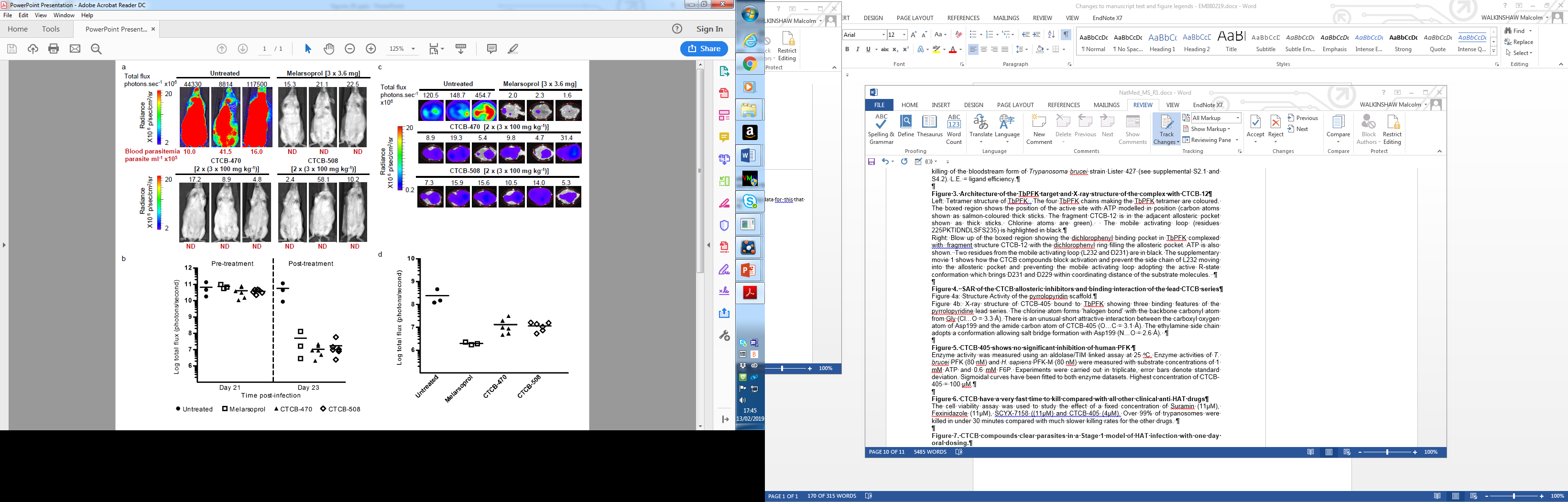


a

b


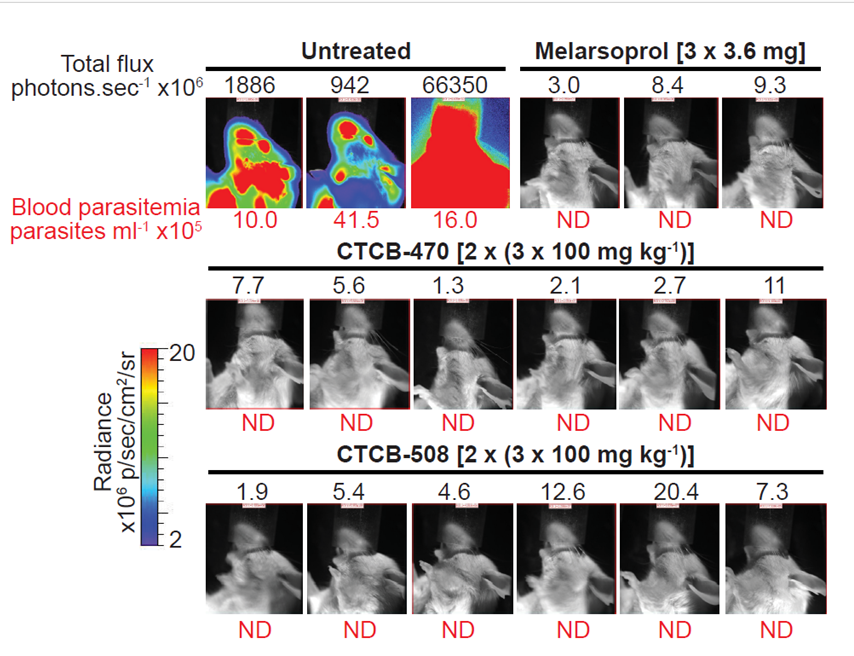


**Supplementary Figure 13. Side head bioluminescence images of mice in the stage 2 model.** T. b. brucei GVR35-infected mice were treated topically with 3 doses of 3.6 mg melarsoprol over 3 days or orally with 6 doses of CTCB compounds (CTCB-470 or CTCB-508) at 100 mg.kg^-1^ over 2 days from day 21 post-infection and were imaged live at day 23 post-infection (n=3 for untreated and melarsoprol-treated mice and n=6 for CTCB-470 and CTCB-508-treated mice). Bioluminescence (total flux in photons per second, above image) from the head and blood parasitaemia (in parasites.ml^-1^, below image) for each mouse (from Figure 7b in the main paper) is shown.

**Supplementary Tables**

|  | TbPFK | CTCB-405 (0.5 x IC_50_ ) | CTCB-405 (1x IC_50_) | CTCB-405( 2 x IC_50_) |
| --- | --- | --- | --- | --- |
| Michaelis Menten best fit versus ATP |  |  |  |  |
| V_max_ (µmol/min.mg) | 36.4 | 36.5 | 33.8 | 26.6 |
| K_m_ (µM) | 118 | 295 | 140 | 150 |
| Michaelis Menten best fit versus F6P |  |  |  |  |
| V_max_ (µmol/min.mg) | 41.5 | 38.7 | 33.6 | 34.0 |
| K_m_ (µM) | 1010 | 2313 | 1277 | 1608 |

**Supplementary Table 1.** Enzymatic parameters of TbPFK obtained by fitting Michaelis-Menten titrations of ATP and F6P at three different concentrations of CTCB-405 (experimental conditions as for Supplementary Figure 2).

|  | CTCB-12 | CTCB-360 | CTCB-405 |
| --- | --- | --- | --- |
| Resolution range | 96.67 - 3.4 (3.522 - 3.4) | 59.07 - 2.35 (2.434 - 2.35) | 48.02 - 2.75 (2.848 - 2.75) |
| Space group | P 2_1_ 2_1_ 2_1_ | P 2_1_ 2_1_ 2 | P 2_1_ 2_1_ 2 |
| Unit cell (Å) | 115.251 132.513 282.699 | 166.154 150.561 83.993 | 150.584 165.088 83.384 |
| Total reflections | 262408 (17864) | 843594 (39399) | 218212 (10911) |
| Unique reflections | 60210 (5917) | 88344 (8711) | 50992 (5118) |
| Multiplicity | 4.4 (4.1) | 9.5 (8.7) | 4.0 (4.0) |
| Completeness (%) | 99.73 (99.90) | 99.89 (99.77) | 92.98 (94.78) |
| Mean I/sigma(I) | 8.4 (0.7) | 10.8 (1.4) | 9.5 (1.4) |
| Wilson B-factor | 115.05 | 47.96 | 48.68 |
| R-merge (%) | 15.7 (188) | 12.9 (179) | 14.5 (115) |
| Reflections used in refinement | 60183 (5917) | 88331 (8712) | 50974 (5117) |
| Reflections used for R-free | 2968 (281) | 4390 (443) | 2503 (274) |
| R-work (%) | 25.1 (41.9) | 20.55 (30.74) | 23.31 (35.52) |
| R-free (%) | 26.7 (40.70) | 24.68 (33.00) | 27.11 (35.19) |
| Number of non-hydrogen atoms | 28255 | 14693 | 13869 |
| macromolecules | 28111 | 14034 | 13688 |
| ligands | 144 | 152 | 96 |
| solvent |  | 507 | 85 |
| Protein residues | 3645 | 1816 | 1774 |
| RMS(bonds, Å) | 0.007 | 0.014 | 0.010 |
| RMS(angles, ° ) | 1.48 | 1.65 | 1.54 |
| Ramachandran favoured (%) | 90.00 | 96.44 | 95.59 |
| Ramachandran allowed (%) | 8.0 | 3.01 | 3.78 |
| Ramachandran outliers (%) | 1.2 | 0.56 | 0.63 |
| Rotamer outliers (%) | 7.5 | 8.75 | 9.15 |
| Clashscore | 8 | 2.53 | 2.93 |
| Average B-factor | 138.71 | 57.42 | 58.32 |
| macromolecules | 138.66 | 57.67 | 58.45 |
| ligands | 147.10 | 63.84 | 60.51 |
| solvent |  | 48.64 | 35.56 |
| PDB code | 6QU5 | 6QU3 | 6QU4 |

**Supplementary Table 2.** Crystallographic data for X-ray structures of TbPFK complexed with CTCB-12, CTCB-360 and CTCB-405.

| **Glycerol (mM)** | EC_50_ CTCB-405 (µM) | EC_50_ CTCB-470 (µM) | EC_50_ CTCB-508 (µM) |
| --- | --- | --- | --- |
| **0** | **0.27** | **0.23** | **0.30** |
| **0.75** | **0.33** | **0.26** | **0.31** |
| **1.5** | **0.32** | **0.26** | **0.34** |
| **5.0** | **0.45** | **0.31** | **0.44** |

**Supplementary Table 3.** Glycerol, added at different concentrations to cultures of bloodstream-form T. b. brucei Lister 427 in regular glucose-containing growth medium has little effect on the EC_50_ values of the CTCB compounds.

|  | T. b. rhodesiense | T. b. gambiense | T. b. brucei (at Edinburgh) |
| --- | --- | --- | --- |
|  | EC_50_ μM | EC_50_ μM | EC_50_ μM |
| CTCB-405 | 0.25 | 0.19 | 0.37 |
| CTCB-470 | 0.28 | 0.14 | 0.31 |

**Supplementary Table 4.** **EC_50_ values of CTCB compounds against the different T. brucei subspecies.**

|  | **CTCB-405** | |
| --- | --- | --- |
| **Dose (mg/kg)** | Predicted plasma concentration @2hr after final dose (µM) | Experimental plasma concentration measured @2hr after final dose (µM) |
| **3 x 50** | 4.1 | 6.8 ± 2.1 |
| **3 x 100** | 8.3 | 9.8 ± 3.1 |

**Supplementary Table 5.** **Comparison of predicted and measured plasma concentrations of CTCB-405 for a multiple dosing regimen.** Three doses of the compound were administered via oral gavage, with a 2 hour interval between each dose. Plasma samples were harvested 2 hours after the third and final dose was delivered.

|  | Alog P | Measured logP | Calc pKa | Measured pKa |
| --- | --- | --- | --- | --- |
| CTCB-405 | 3.57 | 3.29 | 8.32 | 8.09 |
| CTCB-531 | 3.58 | 3.42 | 8.38 | 8.65 |

**Supplementary Table 6. Comparison of calculated and measured logP and pKa values of selected CTCB compounds.**

**Supplementary References**

1. P. M. Fernandes et al., The kinetic characteristics of human and trypanosomatid phosphofructokinases for the reverse reaction. Biochem J **476**, 179-191 (2019).

2. M. A. Wear, M. W. Nowicki, E. A. Blackburn, I. W. McNae, M. D. Walkinshaw, Thermo-kinetic analysis space expansion for cyclophilin-ligand interactions - identification of a new nonpeptide inhibitor using Biacore T200. FEBS open bio **7**, 533-549 (2017).

3. W. Kabsch, Xds. Acta crystallographica. Section D, Biological crystallography **66**, 125-132 (2010).

4. M. D. Winn et al., Overview of the CCP4 suite and current developments. Acta crystallographica. Section D, Biological crystallography **67**, 235-242 (2011).

5. E. Myburgh et al., In vivo imaging of trypanosome-brain interactions and development of a rapid screening test for drugs against CNS stage trypanosomiasis. PLoS neglected tropical diseases **7**, e2384 (2013).

6. B. Raz, M. Iten, Y. Grether-Buhler, R. Kaminsky, R. Brun, The Alamar Blue assay to determine drug sensitivity of African trypanosomes (T.b. rhodesiense and T.b. gambiense) in vitro. Acta tropica **68**, 139-147 (1997).

7. E. Pineda et al., Glycerol supports growth of the Trypanosoma brucei bloodstream forms in the absence of glucose: Analysis of metabolic adaptations on glycerol-rich conditions. PLoS pathogens **14**, e1007412 (2018).

8. J. Kovarova et al., Gluconeogenesis using glycerol as a substrate in bloodstream-form Trypanosoma brucei. PLoS pathogens **14**, e1007475 (2018).

9. M. T. van der Merwe et al., Lactate and glycerol release from adipose tissue in lean, obese, and diabetic women from South Africa. The Journal of clinical endocrinology and metabolism **86**, 3296-3303 (2001).

10. E. Hagstrom-Toft, S. Enoksson, E. Moberg, J. Bolinder, P. Arner, Absolute concentrations of glycerol and lactate in human skeletal muscle, adipose tissue, and blood. The American journal of physiology **273**, E584-592 (1997).

11. I. Kralova, D. J. Rigden, F. R. Opperdoes, P. A. Michels, Glycerol kinase of Trypanosoma brucei. Cloning, molecular characterization and mutagenesis. European journal of biochemistry **267**, 2323-2333 (2000).

12. W. J. Herbert, W. H. Lumsden, Trypanosoma brucei: a rapid "matching" method for estimating the host's parasitemia. Experimental parasitology **40**, 427-431 (1976).

13. A. P. McLatchie et al., Highly sensitive in vivo imaging of Trypanosoma brucei expressing "red-shifted" luciferase. PLoS neglected tropical diseases **7**, e2571 (2013).

14. J. M. Atouguia, F. W. Jennings, M. Murray, Successful treatment of experimental murine Trypanosoma brucei infection with topical melarsoprol gel. Transactions of the Royal Society of Tropical Medicine and Hygiene **89**, 531-533 (1995).

15. M. D. Walkinshaw, S. N. Pettit, A. Highton, I. W. McNae, WIPO, Ed. (2019), chap. WO/2019/106368.
