# Supplementary material for: Fast acting allosteric phosphofructokinase inhibitors block trypanosome glycolysis and cure acute African trypanosomiasis in mice
Source: Nat Commun. 2021 Feb 16;12:1052. doi: 10.1038/s41467-021-21273-6 (PMC7887271; doi:10.1038/s41467-021-21273-6)
Supplement: Supplementary file 3 — Description of Additional Supplementary Files [file 41467_2021_21273_MOESM3_ESM.pdf]

Description of Additional Supplementary Files

File Name: Supplementary Movie 1

Description: Shows the active site of T. brucei Phosphofructokinase with the substrate molecules ATP and fructose 6-phosphate (F6P) and magnesium ion (shown as a small green sphere) modelled in place. The movie morphs together the X-ray structures of the inactive T-form and the active R-state form. The T- state shows the catalytically important aspartate residues (D229 and D231) remote from the magnesium, while in the R-state structure the D229 and D231 have moved to positions where they facilitate phosphoryl transfer between ATP and F6P. When the allosteric pocket adjacent to the active site is occupied by one of the inhibitor molecules (in this case CTCB-405 shown in brown space filling and sticks), the enzyme is trapped in the inactive T-conformation.

File Name: Supplementary Movie 2

Description: Compares the X-ray structures of T. brucei Phosphofructokinase (TbPFK) with the P-isomer of human Phosphofructokinase (hPFK-P) to show the unique binding pocket for the CTCB series of inhibitors that is only present in the parasite structure. The X-ray structure of TbPFK (surface representation in green) is complexed with CTCB-405 (sticks). This is overlaid with the X-ray structure of hPFK-P (yellow) which has a well conserved active site, however the adjacent allosteric pocket is not present making it impossible for CTCB-405 to bind.

Gaaiho PDF Trial
www.gaaiho.com
