# Supplementary material for: Fast acting allosteric phosphofructokinase inhibitors block trypanosome glycolysis and cure acute African trypanosomiasis in mice
Source: Nat Commun. 2021 Feb 16;12:1052. doi: 10.1038/s41467-021-21273-6 (PMC7887271; doi:10.1038/s41467-021-21273-6)
Supplement: Supplementary file 6 — Reporting Summary [file 41467_2021_21273_MOESM6_ESM.pdf]

nature research

Corresponding author(s): Malcolm Walkinshaw
Simon Petit

Last updated by author(s): Oct 23, 2020

Reporting Summary

Nature Research wishes to improve the reproducibility of the work that we publish. This form provides structure for consistency and transparency in reporting. For further information on Nature Research policies, see our Editorial Policies and the Editorial Policy Checklist.

Statistics

For all statistical analyses, confirm that the following items are present in the figure legend, table legend, main text, or Methods section.

n/a	Confirmed	
☐	☒	The exact sample size (*n*) for each experimental group/condition, given as a discrete number and unit of measurement
☐	☒	A statement on whether measurements were taken from distinct samples or whether the same sample was measured repeatedly
☒	☐	The statistical test(s) used AND whether they are one- or two-sided<br>*Only common tests should be described solely by name; describe more complex techniques in the Methods section.*
☒	☐	A description of all covariates tested
☒	☐	A description of any assumptions or corrections, such as tests of normality and adjustment for multiple comparisons
☐	☒	A full description of the statistical parameters including central tendency (e.g. means) or other basic estimates (e.g. regression coefficient) AND variation (e.g. standard deviation) or associated estimates of uncertainty (e.g. confidence intervals)
☒	☐	For null hypothesis testing, the test statistic (e.g. *F*, *t*, *r*) with confidence intervals, effect sizes, degrees of freedom and *P* value noted<br>*Give P values as exact values whenever suitable.*
☒	☐	For Bayesian analysis, information on the choice of priors and Markov chain Monte Carlo settings
☒	☐	For hierarchical and complex designs, identification of the appropriate level for tests and full reporting of outcomes
☒	☐	Estimates of effect sizes (e.g. Cohen's *d*, Pearson's *r*), indicating how they were calculated

Our web collection on statistics for biologists contains articles on many of the points above.

Software and code

Policy information about availability of computer code

Data collection	X-ray data were processed with XDS and AIMLESS.
Data analysis	Initial phases were obtained by molecular replacement using the program PHASER and the structure refined by REFMAC and COOT as implemented in the CCP4 software suite. Graph fitting was carried out using Kaleidagraph 4.0

For manuscripts utilizing custom algorithms or software that are central to the research but not yet described in published literature, software must be made available to editors and reviewers. We strongly encourage code deposition in a community repository (e.g. GitHub). See the Nature Research guidelines for submitting code & software for further information.

Data

Policy information about availability of data

All manuscripts must include a data availability statement. This statement should provide the following information, where applicable:

- Accession codes, unique identifiers, or web links for publicly available datasets
- A list of figures that have associated raw data
- A description of any restrictions on data availability

Data availability Structural X-ray data for protein-ligand complexes are available in the Protein Database with accession numbers 6QU5, 6QU3 and 6QU4

Field-specific reporting

Life sciences study design

All studies must disclose on these points even when the disclosure is negative.

Sample size	The number of animals assigned to each treatment group was selected in compliance with animal ethics regulations, so as to provide sufficient statistical power to discern significant differences.<br>For the T. b. brucei GVR35 mouse model sample sizes were determined using a power calculation with data (means and standard deviations) from previous published experiments (e.g. Khare et al and Myburgh et al). Based on these experiments a compound that shows trypanocidal activity to peripheral parasites should result in a 10-fold decrease in body bioluminescence after treatment compared to untreated controls. The significance level is set at 5%, power at 80% and a two-sided test selected because we cannot predict whether a compound may reduce or exacerbate the infection.
Data exclusions	No data were excluded from analyses.
Replication	All experiments involving animals or cells/parasites used a minimum of 3 independent biological replicates and, in the case of cell based assays 3 technical replicates. All experiments were able to be reliably reproduced. T. b. brucei GVR35 mouse experiments were not replicated for the doses shown in the manuscript
Randomization	Age and sex matched animals were randomly assigned to experimental and control groups.
Blinding	Blinding was not possible for animal experiments. The work required specific expertise in working with T.b. brucei and running the imaging model. All animal infections, treatments and imaging was performed by the same researchers. Blinding between the untreated, melarsoprol-treated and compound-treated groups was not possible due to the different routes of treatment for the control and test compounds.

Reporting for specific materials, systems and methods

We require information from authors about some types of materials, experimental systems and methods used in many studies. Here, indicate whether each material, system or method listed is relevant to your study. If you are not sure if a list item applies to your research, read the appropriate section before selecting a response.

Materials & experimental systems

n/a	Involved in the study
☒	☐ Antibodies
☐	☒ Eukaryotic cell lines
☒	☐ Palaeontology and archaeology
☐	☒ Animals and other organisms
☒	☐ Human research participants
☒	☐ Clinical data
☒	☐ Dual use research of concern

Methods

n/a	Involved in the study
☒	☐ ChIP-seq
☒	☐ Flow cytometry
☒	☐ MRI-based neuroimaging

Eukaryotic cell lines

Policy information about cell lines

Cell line source(s)	ECACC 85011430
Authentication	STR profiling
Mycoplasma contamination	Tested negative for mycoplasma contamination
Commonly misidentified lines (See ICLAC register)	N/A

Animals and other organisms

Policy information about studies involving animals; ARRIVE guidelines recommended for reporting animal research

Laboratory animals	6-8 weeks old female CD-1 mice were used for all non-GVR-35 trials. All T.b. brucei GVR35 animal experiments were done with female CD1 mice that were 8-9 weeks old at the time of infection. For housing of mice, the temperature was set at 21°C, humidity set for 50-55% and light/dark cycle set at 12 hours on, 12 hours off with light between 7 am and 7 pm.
Wild animals	No wild animals were used in this study
Field-collected samples	No field collected animals were used in this study
Ethics oversight	All experiments were carried out in accordance with the United Kingdom Animals (Scientific Procedures) Act

(1986) under Home Office regulations following ethical approval from the local AWERB. Studies were performed in SPF facilities at the University of Edinburgh under licence PPL 70/8102 and at the University of Glasgow under PPL 60/4442. The experiments were approved by the local ethics committees of the respective universities.

Note that full information on the approval of the study protocol must also be provided in the manuscript.
